# Effect of Chloride Ions Concentrations to Breakdown the Passive Film on Rebar Surface Exposed to L-Arginine Containing Pore Solution

**DOI:** 10.3390/ma14195693

**Published:** 2021-09-30

**Authors:** Jitendra Kumar Singh, Soumen Mandal, Han-Seung Lee, Hyun-Min Yang

**Affiliations:** 1 Innovative Durable Building and Infrastructure Research Center, Hanyang University, 55 Hanyangdaehak-ro, Sangrok-gu, Gyeonggi-do, Ansan-si 15588, Korea; jk200386@hanyang.ac.kr; 2 Intelligent Construction Automation Center, Kyungpook National University, 80, Daehak-ro, Buk-gu, Daegu 41566, Korea; sou.chm@gmail.com; 3 Department of Architectural Engineering, Hanyang University, 55 Hanyangdaehak-ro, Sangrok-gu, Gyeonggi-do, Ansan-si 15588, Korea

**Keywords:** steel rebar, concrete pore solution, passive film, corrosion, electrochemical impedance spectroscopy, scanning electron microscopy

## Abstract

In the present study, 0.115 M L-arginine (LA) has been used as an eco-friendly inhibitor in simulated concrete pore solutions (SP-0) in order to form passive films on a steel rebar–solution interface until 144 h. Hence, 0.51 (SP-1) and 0.85 M NaCl (SP-2) were added in LA containing SP-0 solution to breakdown the passive film and to initiate corrosion reactions. The electrochemical results show that the charge transfer resistance (*R_ct_*) of steel rebar exposed to SP-1 and SP-2 solutions increased with respect to immersion periods. The sample exposed to the SP-2 solution initiated the corrosion reaction at the steel rebar–solution interface after 24 h of NaCl addition and formed pits; on the other hand, the sample without NaCl added, i.e., SP-0, showed agglomeration and dense morphology of corrosion products.

## 1. Introduction

Premature distress of reinforced concrete (RC) results from poor workmanship, lack of maintenance, atmospheric effects, and the ingress of chloride ion (Cl^−^) into concrete. Moreover, Cl^−^ ions are the most affecting factor for the distress of RC structures [1]. This ion perturbs passive oxide films, which formed onto the embedded rebar in RC and accelerated deterioration via the formation of intermediate corrosion products. Most of the bridges located in coastal areas or those immersed in sea water are prone to corrosion. In this case, Cl^−^ ions act as catalysts to enhance corrosion reactions of embedded rebar [2]. Therefore, inhibitors are used as alternatives for delaying the onset of corrosion due to their unequivocal advantages, i.e., they are convenient, inexpensive, available, and highly corrosion resistant. It was proven that nitrite based inhibitors are very effective for combating the corrosion of steel rebar, however, it has been debarred owing to its carcinogenicity and toxicity [3].

There are many controversies regarding the threshold values of chloride ions for de-passivation or the initiation of steel rebar corrosion exposed to concrete, mortar, and pore solution conditions. Alonso et al. [4] have explained that there is no unique or fixed value of chloride threshold for active corrosion of steel rebar embedded in concrete because it depends on several factors such as water/cement ratio, relative humidity, temperature, blended materials, concrete mix proportions, cement type, tricalcium aluminate content in cement, steel chemistry and surface conditions, source of chloride, and types of inhibitors [4]. However, Hausmann and Gouda have determined that the ratio of 0.6 [Cl^−^]/[OH^−^] is the chloride threshold for steel rebars immersed in pore solutions [5,6]. Vassie has derived the chloride threshold value of bridges in UK, and it numberd from 0.2 to 1.5% by weight of the cement [7]. Moreover, the British standard limit for chloride threshold measures up to 0.1% for pre-stressed concrete and less than 0.4% for the RC structures [8]. The acceptable ranges of chloride threshold value are from 0.2 to 0.4% by weight of the cement used to predict the corrosion-free life of a concrete structure [9,10,11,12,13]. The chloride threshold value can be increased if the pH of a concrete pore solution increased and is attributed to an increase in hydroxyl ion [14,15]. The chloride threshold values can be deterred based on the types of salt present in the service conditions of the concrete [16].

Xu et al. [17] have determined the chloride threshold values of calcium nitrite (Ca(NO_2_)_2_), zinc oxide (ZnO), and *N*,*N*′-dimethylaminoethanol (DMEA) inhibitors in saturated calcium hydroxide solution, and they have found that inhibitors had a marginal effect on increasing the chloride threshold value [17]. The amino acid based corrosion inhibitor, i.e., aspartate, has increased the threshold chloride content by adsorbing onto the steel surface [18]. The aspartate has chelating characteristics; therefore, it shows inhibitive properties through a negative charge repulsion by a non-adsorbed carboxylate group. Two percent ginger extract has improved the chloride threshold values of steel rebar from 0.02 mol/L to 0.08 mol/L in simulated concrete pore solutions by forming a carbonaceous organic film, which retards cathodic and anodic reactions [19]. The chloride threshold value of *N*,*N*′-dimethylaminoethanol inhibitor has been found to be 0.4 [Cl^−^]/[OH^−^], which can be increased by increasing the concentration of inhibitor [20]. Nihali et al. [21] have studied the effect of Na_3_PO_4_ inhibitor on the chloride threshold in saturated Ca(OH)_2_ solutions, and they have found that, once the inhibitor was added, the chloride threshold value increased from 0.6 to 1.5 and is attributed to the formation of iron phosphate Fe_3_(PO_4_)_2_·8H_2_O as passive films [21].

There are many eco-friendly corrosion inhibitors for the mitigation of steel corrosion in acidic conditions, i.e., HCl and H_2_SO_4_ [22,23,24], but they are scarcely available on the steel rebar corrosion embedded in concrete or exposed to simulated concrete conditions added with NaCl. The chloride threshold of some nitrite-based inhibitor has been studied, and such types of inhibitor are hazardous; therefore, they have been banned by USA and European countries [3]. However, there is little effort made by the researchers with respect to investigating the chloride threshold of eco-friendly corrosion inhibitors [25,26]. While we have described the effect of some eco-friendly inhibitors with respect to retarding steel rebar corrosion in the aforementioned paragraphs, none of the literature investigated or explained the chloride threshold of L-arginine (LA) containing inhibitors with exposure periods in simulated concrete pore (SP) solutions. In our earlier studies, we have found that 0.115 M LA exhibited 96% corrosion inhibition efficiency in 0.17 M NaCl added simulated concrete pore solutions [27]. Therefore, in the present study, we have selected 0.115 M LA for chloride threshold determination. We have kept a steel rebar in SP + 0.115 M LA for 144 h at different concentrations, i.e., a certain amount of NaCl from 0.51 to 0.85 M has been added into the solution to breakdown the passive film and initiate the corrosion reaction. Different electrochemical studies have been performed up to 168 h after the addition of NaCl. The characterizations of passive/oxide films formed onto the steel rebar are studied by scanning electron microscopy (SEM) after 168 of exposure in SP + 0.115 M LA (SP-0), SP + 0.115 M LA + 0.51 M NaCl (SP-1), and SP + 0.115 M LA + 0.85 M NaCl (SP-2) solutions.

## 2. Materials and Methods

### 2.1. Materials

In the present study, a 16 mm diameter and 10 mm thick plain carbon steel rebar possessing chemical compositions shown in Table 1 was determined by X-ray fluorescence (XRF, Rigaku, Tokyo, Japan). The steel rebars were polished with emery paper measuring 220 to 2000 in size. It is important to induce a mirror finished surface on the steel rebar; therefore, they were polished with alumina slurry (0.5 μm particle size). These steel rebars were used for electrochemical studies as well as other characterizations to assess reproducibility and to obtain an average result.

The electrochemical studies were carried out in simulated concrete pore (SP) solutions. The chemical composition for the synthesis of this solution was described in our earlier published work [28,29,30] and others [31,32] where NaOH, KOH, and CaO were used. In this solution, SP was filtered with Whatman paper where insoluble CaO was removed. The amount of 0.115 M L-arginine (LA, purchased from Sigma-Aldrich, Seoul, Korea) was dissolved in the SP solution. The details about LA can be found elsewhere [27,33]. The passivation of steel rebars was performed by keeping them in SP + 0.115 M LA solution for 144 h. After that, different amounts of NaCl were added to determine the chloride threshold of the passive film. The details of the solution composition are shown in Table 2.

### 2.2. Electrochemical Studies

Electrochemical studies of steel rebars were performed on a 0.78 cm^2^ polished surface. Initially, the rebar samples were kept in SP + 0.115 M LA solution, i.e., SP-0 was kept for 144 h to ensure that a uniform passive film has been formed. Thereafter, different amounts, i.e., 0.51 M and 0.85 M NaCl, were added to the solutions in order to breakdown the passive film and to determine the chloride threshold. The samples were kept in the solution for 30 min to achieve a stabilized open circuit potential (OCP) prior to start the electrochemical impedance spectroscopy (EIS) studies. EIS was performed by three electrode systems where the steel rebar functioned as the working electrode (WE), saturated calomel electrode (SCE), and reference electrode (RE) and platinum wire functioned as the counter electrode (CE).

The assessment for the nucleation, growth, and breakdown of passive film formed at steel rebar–solution interface was carried out by EIS using Potentiostat (VersaSTAT (Princeton applied Research, Oak Ridge, TN, USA)). A 10 mV amplitude of sinusoidal voltage varying its frequencies between 100 kHz and 10 mHz was imposed on the exposed surface of the steel rebars at their OCP. Three steel rebar samples were exposed in the solutions, and EIS tests were performed for all the samples. The mean values of the raw data were computed and are shown in the manuscript.

### 2.3. Analysis of the Passive Film and Corrosion Products

After 168 h of exposure in different solutions, the morphology of the passive film/corrosion products was examined by energy dispersive X-ray (EDS) analysis that was complementarily available to scanning electron microscopy (SEM, MIRA3, TESCAN, Brno, Czech Republic) at 15 kV. The steel surface was rinsed with distilled water prior to taking the SEM images to ascertain the removal of deposit salts. The other details about characterization techniques are described in our earlier published papers [29,30].

## 3. Results and Discussion

### 3.1. Electrochemical Studies

#### 3.1.1. Measurement of Open Circuit Potential (OCP)

**Figure 1:**
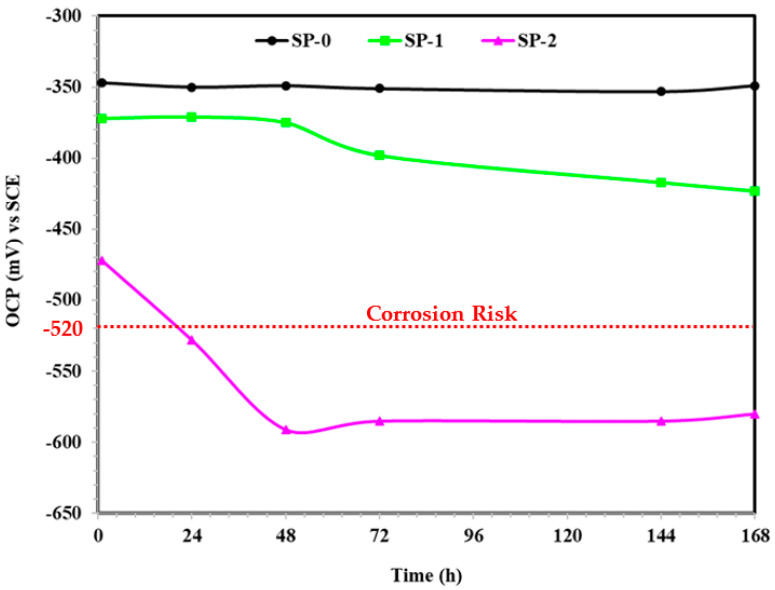
OCP measurement with immersion time.

The OCP measurement results are shown in Figure 1. The OCP of the passivated steel rebar stabilized by adsorbing the LA at the surface, which formed the Fe–Zwitterion complex that protects rebar from corrosion [34]. However, once 0.51 M NaCl was added, i.e., SP-1, the OCP was found to be active compared to SP-0, and it is gradually decreased after 48 h of NaCl addition. As the amount of NaCl increased, i.e., SP-2, the OCP became more negative (active) compared to SP-0 and SP-1, and this is attributed to the breakdown of the passive layer. It is found that up to 0.85 M NaCl, i.e., SP-2, there is no risk of steel rebar corrosion during 1 h of addition because the OCP of this sample is higher at −520 mV vs. SCE. It is suggested by other researchers that if the OCP of steel rebar is falls down to −520 mV vs. SCE, then it is considered as a risk of corrosion when exposed to chloride containing solutions [16,20,35,36,37]. It can be observed from Figure 1 that as the immersion durations were extended up to 24 h, the OCP of steel rebar immersed in SP-2 solution shifted towards the active direction and was lower than −520 mV vs. SCE. Thus, it can be suggested that the chloride threshold of steel rebar exposed to SP + 0.115 M LA solution after 24 h of exposure is 0.85 M NaCl. Moreover, the OCP results of the steel rebar immersed in SP-1 solution up to 168 h of exposure suggest that there is no risk of corrosion. The OCPs of steel rebars exposed to NaCl added solutions, i.e., SP-1 and SP-2, stabilized after 72 h, which is attributed to the complex reaction where Cl^−^ ions form Cl-(Zwitterion)-Fe complex. The LA is fixed in the present study; thus, it required a fixed amount of NaCl to form the Cl-(Zwitterion)-Fe complex, which adsorbed onto the rebar surface. However, once the NaCl amount increased beyond the optimum amount of LA, the Cl^−^ ions locally attacked and initiated corrosion reactions at the surface of the steel rebar. Thus, a lower OCP was found at extended periods of exposure.

#### 3.1.2. EIS with Time

**Figure 2:**
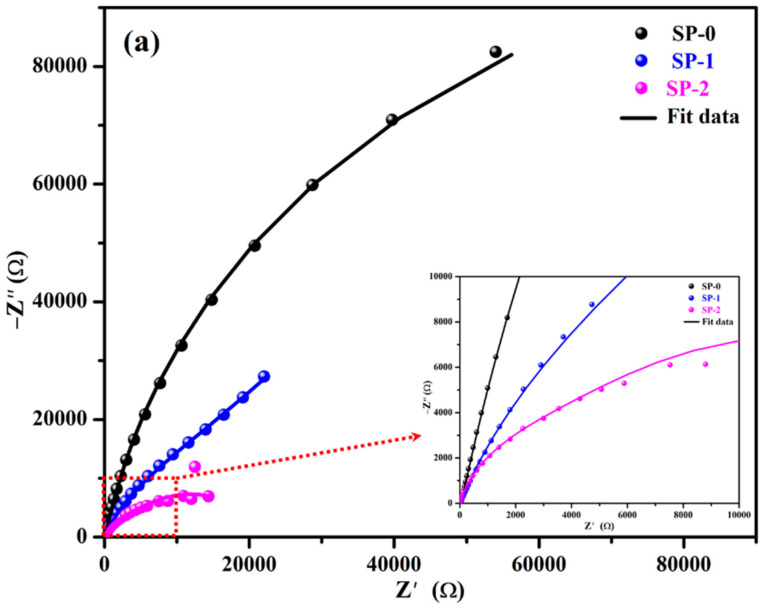

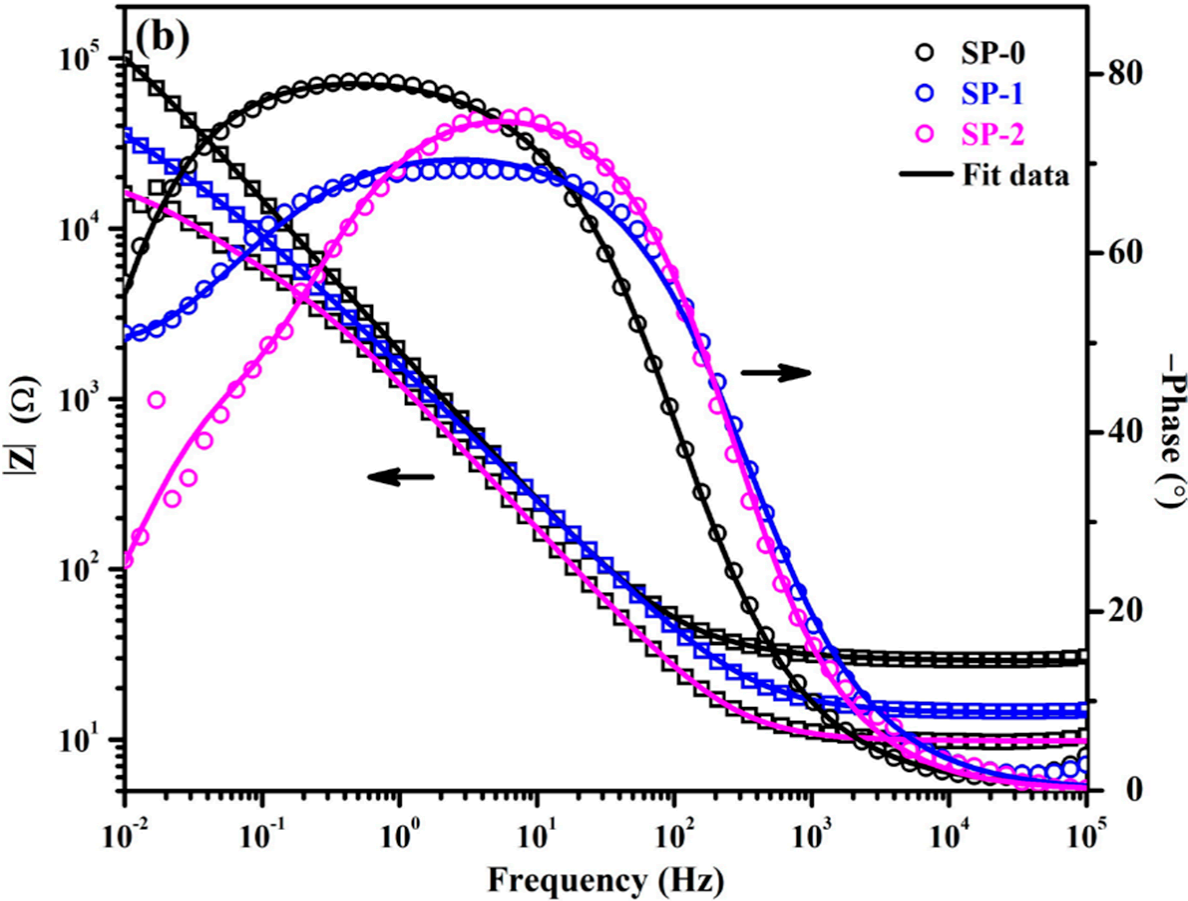
EIS plots for (**a**) Nyquist and (**b**) Bode after 1 h of immersion.

**Figure 3:**
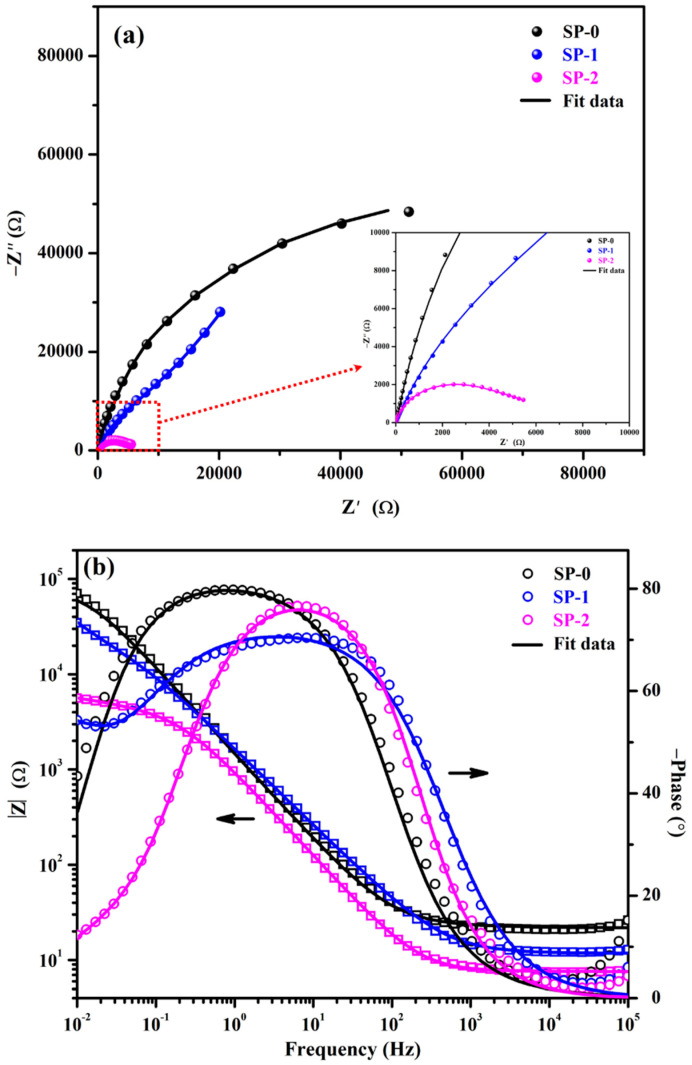
EIS plots for (**a**) Nyquist and (**b**) Bode spectra after 48 h of exposure.

**Figure 4:**
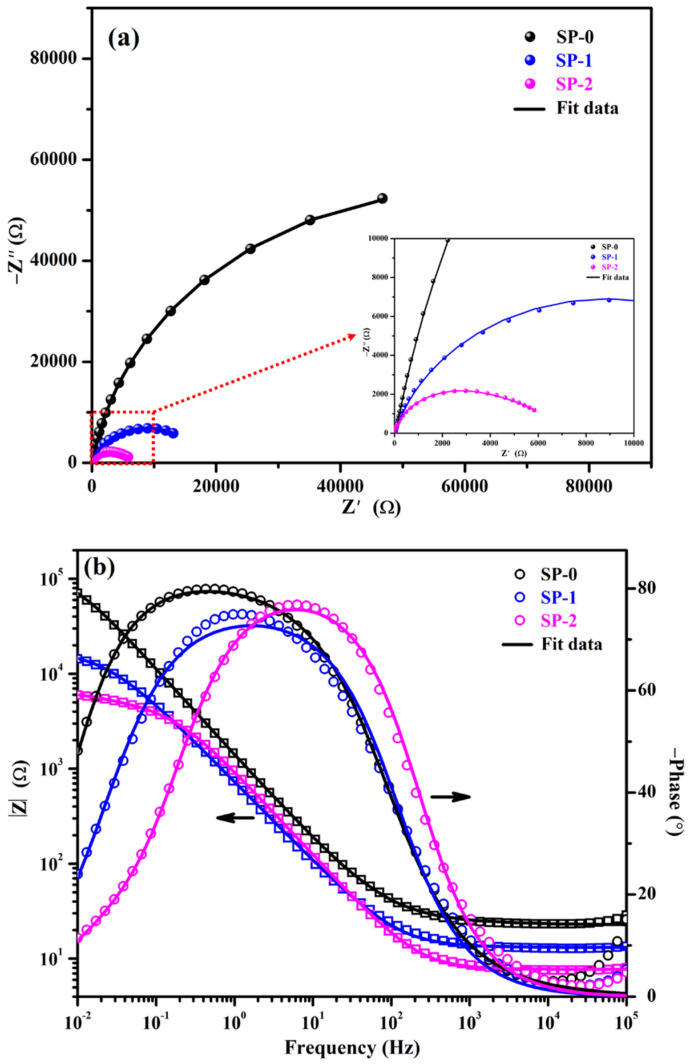
EIS plots for (**a**) Nyquist and (**b**) Bode spectra after 168 h of exposure.

The Nyquist plots of steel rebars with exposure periods are shown in Figure 2, Figure 3 and Figure 4. The Nyquist plots of steel rebar after 1 h of exposure to the SP-0 solution exhibited the highest magnitude (Figure 2a), revealing the formation of adherent and stable film. This film might consist of a Zwitterion–Fe complex. On the contrary, once NaCl was added, there was a significant reduction in magnitude of Nyquist plots (Figure 2a) due to the attack of Cl^−^ ions on the passive film, i.e., Zwitterion–Fe complex [20]. This result correlates with OCP plots where the OCP difference of steel rebar was more than 120 mV vs. SCE between SP-0 and SP-2.

The modulus Bode plots of steel rebars after 1 h of immersion are illustrated in Figure 2b. The total impedance of steel rebar immersed in the SP-0 solution measured the highest at 0.01 Hz followed by SP-1 and SP-2. The impedance values of NaCl added solution decreased gradually once the amount increased, suggesting that the formed passive film or adsorbed Zwitterion-Fe complex started to perturb. It is well known that once the chloride concentration increased, the deterioration of passive film increased [17]. However, it is observed from Figure 2b that there are significant differences in the total impedance of samples immersed in different solutions, which suggests that after addition of different amounts of NaCl during 1 h of exposure, Cl^−^ ions could break the passive film. There is a possibility that once the exposure duration is extended, the total impedance would decrease significantly. Thus, the corrosion characteristics of samples were studied over extended periods.

The Bode phase angles of steel rebars after 1 h of immersion are depicted in Figure 2b. It is observed that the steel rebar immersed in SP-0 solution exhibited two times more constants: one from 2 to 0.08 Hz at −80° and another at lower frequencies, i.e., 0.01 Hz at −56°, which is attributed to formation of strong passive films, i.e., Zwitterion–Fe complex. Alternatively, once 0.51 M NaCl was added in SP + 0.115M LA, i.e., SP1, the maxima shifted at a lower angle, i.e., −70° compared to SP-0. However, broadening from the middle to low studied frequencies revealed that the passive film’s properties were retained. This means that 0.51 M NaCl is not enough to break the strong passive film. However, the steel rebar exposed to SP-2 solution exhibited shifting in terms of the phase angle maxima toward the middle frequency, and the asymmetric capacitive loop becomes sharp. The shifting of the capacitive loop at −75° on 8 Hz revealed that the passive film became weak. Thus, there is probability that if exposure periods increased, the passive films would start to deteriorate, i.e., the initiation of corrosion phenomena occurs. In subsequent paragraphs, we have studied the effect of Cl^−^ ions on deterioration of passive films over extended periods of exposure.

The EIS spectra of the steel rebar immersed in different solutions after 48 h are shown in Figure 3. The magnitude of Nyquist plots of all samples decreased (Figure 3a) compared to 1 h of immersion revealing the breakdown of passive film. However, there is no significant difference in the magnitude reduction in Nyquist plots of SP-0 and SP-1, while SP-2 decreased significantly. This result suggests that SP-0 and SP-1 retained their passive properties, but SP-2 started to deteriorate. The EIS results show that passive films are initially very strong, adherent, and protective, which required 0.85 M NaCl and 48 h to breakdown the passive film and to initiate the corrosion phenomena at the steel rebar–solution interface. Moreover, the reduction in magnitude of Nyquist of NaCl added samples suggest that Cl^−^ ions are attacking onto the passive film and weakening the passive film. Initially, Cl^−^ ions interact with –NH_3_^+^ (Zwitterion) in the Zwitterion–Fe complex, which resulted in the formation of weak bonds between Zwitterion and Fe [18,27,34]. Thus, a Cl-(Zwitterion)-Fe complex is formed rather than a Zwitterion-(Cl)-Fe complex. In the case of high amounts of NaCl, i.e., SP-2, Cl^−^ ions are appear in significant amounts, and they are able to form a Cl-(Zwitterion)-Fe complex, but some unreacted Cl^−^ ions ingress towards the steel surface and initiate corrosion reactions. This observation is well-corroborated with OCP results where, after 48 h of exposure, it activated and initiated corrosion reactions.

The Bode modulus results after 48 h of immersion are shown in Figure 3b. There is no significant difference in the total impedance of steel rebars immersed in the SP-1 solution compared to 1 h revealing that 0.51 M NaCl is not able to break the passive film immersed in the SP + 0.115 M LA solution. However, in the case of SP-2, the total impedance was reduced by three times than 1 h, which suggests that corrosion initiation started to occur after 48 h of immersion. This finding reveals that the passive film formed in SP + 0.115 M NaCl solution required 0.85 M NaCl and 48 h to breakdown the passive film and to initiate corrosion. Meanwhile, Cl^−^ ions in this solution also formed a Cl-(Zwitterion)-Fe complex, but some unreacted Cl^−^ ions during complex formation locally attacked the steel rebar and initiated corrosion reactions.

The steel rebar immersed in different solutions up to 48 h and the phase Bode plots results are shown in Figure 3b. The steel rebar immersed in SP-0 solution has maintained its phase maxima from 5 to 0.10 Hz at −80°, revealing the protective properties of the film. Broadening in the phase angle maxima of steel rebar immersed in SP-1 solution is observed from 31 to 0.5 Hz at −70°. This result suggests that steel rebar immersed in SP-1 solution retained its protective properties even after 48 h owing to the formation of Cl-(Zwitterion)-Fe complex. Alternatively, there is sharp asymmetric capacitive loop observed in the SP-2 solution exposed sample at −75° on 6 Hz. There is an interesting observation found for the rebar immersed in SP-2 solution at 0.01 Hz where phase maxima are found to be −12°, while it is −26° after 1 h of exposure. The shifting in maxima towards a lower angle at 0.01 Hz revealed the initiation of corrosion. Thus, significant reduction in total impedance was observed after 48 h of exposure.

The EIS plots after 168 h of immersion are illustrated in Figure 4. The Nyquist plots of samples are shown in Figure 4a. The steel rebar exposed to SP-0 solution retained its properties as observed after 48 h of exposure, which suggests that passive films strengthen with exposure periods in SP + 0.115 M LA solution owing to the formation of a Zwitterion–Fe complex. However, the NaCl added sample, i.e., SP-1, exhibited reductions in magnitude of Nyquist plots than earlier periods of exposure owing to the interaction of Cl^−^ ions with Zwitterion. In this case, electrostatic interactions between Zwitterion and Fe decreased, resulting in weakening of passive films. Alternatively, the steel rebar exposed to the SP-2 solution shows identical magnitudes in Nyquist plots as observed after 48 h of immersion. This result reveals that once the corrosion reaction started after 48 h of exposure, it did not significantly enhance or increase corrosion; rather it maintained the properties of the film.

The total impedances of the samples after 168 h of immersion are shown in Figure 4b. The steel rebars exposed to SP-0 and SP-2 solutions maintained their total impedance obtained after 48 h of exposure, but SP-1 was observed to be slightly decreased. In the case of SP-0, the passive films are strengthened and stabilized due to the surface coverage of the passive film. However, in the case of SP-2, it was found that once the corrosion has started after 48 h of immersion in the presence of high amount of Cl^−^ ions, the Cl-(Zwitterion)-Fe complex stabilized and controlled further corrosion reaction. Therefore, this demonstrated identical impedance by the formation of complexes as observed in earlier exposure periods. However, the steel rebar exposed to SP-1 solution exhibited reduction in total impedance compared to earlier exposure periods, but it is higher than SP-2 owing to weakened Fe-(Zwitterion)-Cl/Cl-(Zwitterion)-Fe complex. The electrostatic interaction between Fe–Zwitterion is weak compared to the Zwitterion–Cl complex because the –COO^−^ group of Zwitterion has lower affinity with Fe compared to the Zwitterion–Cl complex [27,38,39,40].

The results of phase angle Bode plots after 168 h of exposure are shown in Figure 4b. The steel rebar immersed in SP-0 solution maintained its phase angle maxima even after 168 h of exposure around −80° from 2 to 0.08 Hz. This result suggests that steel rebar surface is protected by the adsorbed Zwitterion that formed a protective film, i.e., the passive film strengthened its resistive properties by forming a uniform and homogeneous layer of Zwitterion–Fe complex within the exposure period. Furthermore, the steel rebar immersed in SP-1 solution exhibited phase maxima at −75° on 0.96 Hz, but this asymmetric capacitive loop is sharp; on the other hand, there was broadening in peak from middle to low frequencies after 48 h of exposure. There is an interesting observation where the maxima are found at −24° on 0.01 Hz, and it is much lower than earlier immersion periods. The lowering in maxima and sharpness in the asymmetric capacitive loop indicates that this sample started to reduce the properties of passive film at longer duration of exposure. Since the steel rebar kept in SP-2 solution exhibited phase angle maxima at −76° on 11 Hz, the shifting of maxima in middle frequencies and sharpness in asymmetric capacitive loops indicates the initiation of corrosion reactions. Moreover, the shifting of maxima towards 0.01 Hz at −11° revealed the initiation of corrosion at steel rebar–solution interface. The unreacted Cl^−^ ions with Zwitterion in the solution ingress through the Zwitterion–Fe layer and initiated the corrosion reaction. Thus, the lowest total impedance is found. There is no difference in phase angle maxima of steel rebar immersed in SP-2 solution at 0.01 Hz between 48 h and 168 h, revealing the stabilization of corrosion reaction owing to the formation of a Fe-(Zwitterion)-Cl complex. This result is well corroborated with OCP plots where the OCP of this sample stabilized after 72 h of exposure. There is the possibility that film thickness is high, which stifled the ingress of NaCl towards steel rebar surface. Thus, stabilized impedance is observed after 168 h of exposure.

**Figure 5:**
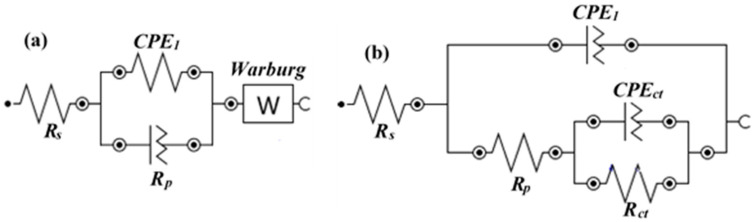
EEC of (**a**) SP-0 and (**b**) SP-1 to SP-2 samples from 1–168 h of exposure.

The electrical equivalent circuit (EEC) has been used to fit EIS data and to extract electrochemical parameters. Figure 5a was used to fit EIS data of the steel rebar exposed to SP-0 solution with different exposure periods where a single circuit is fitted with one time constant and Warburg impedance [41,42]. In this EEC, *R_s_*, *CPE*, and *R_p_* are the solution resistance, constant phase element, and polarization resistance, respectively. The presence of CPE is due to the heterogeneity of the surface. Alternatively, EIS plots of steel rebar immersed in SP-1 and SP-2 solutions are fitted with one EEC containing two times constant, as shown in Figure 5b [43,44,45,46]. In this EEC, *R_ct_* and *CPE_ct_* are the charge transfer resistance and constant phase element for charge transfer, respectively.

The CPE coefficient (*Q_eff_*) can be calculated by Equation (1) with the CPE exponent (*n*) ≠ 1 [47].

(1) $$Q_{eff}=\sin\left(\frac{n\pi }{2}\right)\frac{-1}{Z_{j}(f){\left(2\pi f\right)}^{n}}$$

In Equation (1), *f* is frequency. However, *Q_eff_* becomes capacitance (*C_eff_)* if *n* is 1. Therefore, Equation (1) can be rewritten as follows.

(2) $$Q_{eff}=C_{eff}=\frac{-1}{Z_{j}(f)(2\pi f)}$$

Due to exposure of rebars in solution, surface heterogeneity is found. Thus, *C_eff_* can be determined by the CPE coefficient (*Q*) [48,49,50].

(3) $$C_{eff}=Q^{1/n}R_{S}^{(1-n)/n}$$

The thickness of the passive film (*t_f_*) is calculated on the basis of obtained *C_eff_* as follows [51]:

(4) $$t_{f}=\frac{A\cdot \varepsilon \varepsilon_{0}}{Ceff}$$

where *A* is the surface exposed area of steel rebar, and *ε* and *ε*_0_ are permittivity of steel and permittivity of vacuum, respectively.

**Figure 6:**
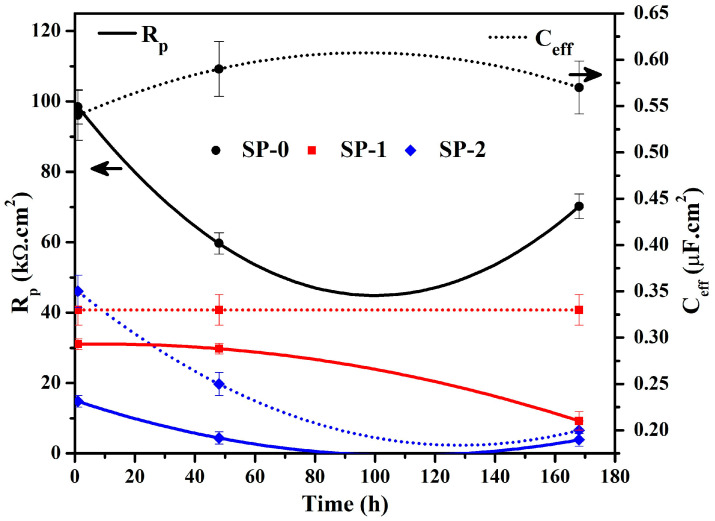
*R_p_* and *C_eff_* in different solutions with time.

The electrochemical parameters are calculated by fitting EIS plots in suitable EEC, and the results are shown in Table 3 and Figure 6. The *R_s_* value of steel rebar exposed to SP-0 solution is higher and found to be 22–28 Ω·cm^2^ compared to NaCl added solutions, as shown in Table 3. Initially L-arginine dissolved in the solution, but once it was exposed to steel rebar, it formed a Zwitterion–Fe complex at the steel rebar–solution interface where all L-arginine might be consumed during the formation of this complex. The solubility of this complex might be significantly lower; thus, the ion concentrations in this solution also becomes lower. Therefore, it shows the highest *R_s_* values compared to the others. Alternatively, once NaCl was added in the solution, the ions’ mobility and concentration increased; thus, lower *R_s_* values were observed. It can be observed from Table 3 that the steel rebar immersed in SP-2 solution exhibits the lowest *R_s_* values compared to the others owing to the highest amount of NaCl in solution. The *R_p_* value of the samples gradually decreased as immersion time increased (Figure 6), and this is attributed to the diffusion of Zwitterion–Fe complex by coordinating with oxygen, which results in Warburg impedance (W) in the SP-0 solution while this is not observed in SP-1 and SP-2 owing to the interaction of Cl^−^ ions with Zwitterion–Fe. This causes the ingress of Cl^−^ ions in SP-1 and SP-2 solutions, which weakens the Zwitterion–Fe complex. In the case of SP-2, Cl^−^ ions appear in significant amounts, which perturb the passive film and initiated corrosion reaction after 48 h of exposure. This result well correlates with the OCP plots (Figure 1). Once the *R_p_* values decreased, *C_eff_* increased with exposure periods. The reduction in *R_p_* values in SP-2 solution is significant up to 48 h, but it is stabilized after 168 h of exposure. Initially, W is lower in SP-0, but it is increased around two times as immersion time increased up to 48 h (Table 3), which suggests that oxygen (from Zwitterion) diffused towards the surface of the steel rebar but decreased after 168 h again (Table 3). It can be observed from Table 3 that *n*_1_ values of all samples after 1 h of exposure is found to be ≥0.8, revealing that the surface becomes homogeneous [45,52]. However, as the exposure periods are extended, the steel rebar exposed to SP-0 and SP-1 solutions attain its values ≥0.8, which suggests that passive films are protective and cannot be affected by aggressive ions in the solution, while it decreased for SP-2. This result suggests that an amount more than 0.51 M NaCl results the passive film breaking and the surface becomes heterogeneous; thus, pitting corrosion can be observed. The *R_ct_* values of steel rebar exposed to SP-1 and SP-2 solutions increased with exposure periods (Table 3). Moreover, SP-1 exhibiting greater *R_ct_* values than SP-2. This is attributed to the formation of protective Cl-(Zwitterion)-Fe complex as passive films, but Cl^−^ ions in SP-2 solution are significant, which perturbs the passive film. The *n_ct_* values of steel rebar exposed to SP-1 and SP-2 solutions increased as immersion periods increased (Table 3). However, in the case of SP-1, *n_ct_* values are close to 0.8, suggesting the formation of homogenous passive film, while SP-2 is found to be at the maximum of 0.65 after 168 h of exposure, revealing its defective and heterogeneous properties. *Q_ct_* values of steel rebar exposed to SP-2 solution are higher than SP-1, revealing the capacitive properties of the film. The thickness of the film with exposure periods increased in NaCl containing samples, and this is attributed to the formation of the Cl-(Zwitterion)-Fe complex, while SP-0 exhibited lower values and stabilized at 35–38 nm (Table 3). There is no NaCl; thus, SP-0 cannot form the above complex. Thus, it reflects lower values.

#### 3.1.3. Potentiodynamic Polarization Studies after 168 h of Exposure in Different Solutions

**Figure 7:**
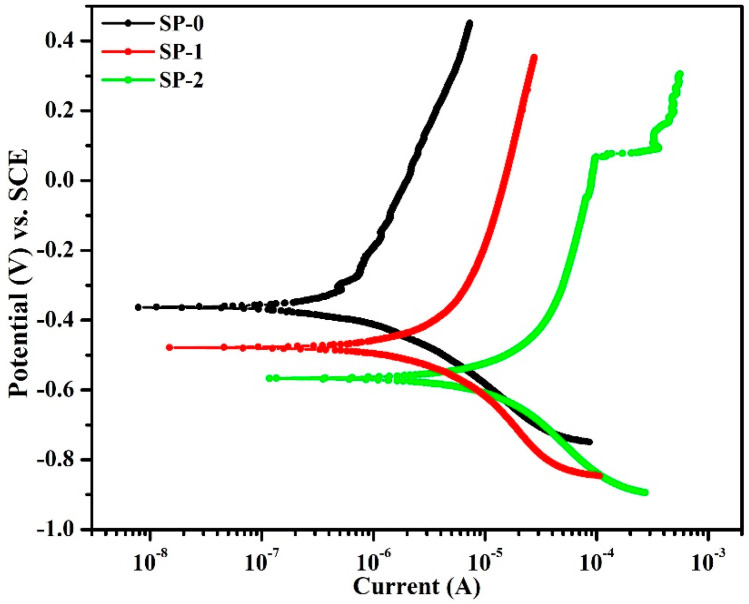
Potentiodynamic polarization after 168 h immersion in different solutions.

The potentiodynamic polarization results of steel rebar after 168 h of immersion are shown in Figure 7. The cathodic curve results indicate oxygen reduction reactions. During cathodic scanning, the Zwitterion–Fe complex reduced, resulting in increased cathodic current density. The steel rebar immersed in SP-2 solution exhibits the highest cathodic current due to the development of a defective passive layer by Cl^−^ ions, which weakens the Cl-(Zwitterion)-Fe complex. Alternatively, during anodic scanning, the current density of SP-0 sample was found to be lowest followed by SP-1 and SP-2. The anodic currents of steel rebar exposed to SP-1 and SP-2 solutions are higher than SP-0 due to the presence of Cl^−^ ions, which destabilized the Zwitterion–Fe complex and enhanced corrosion reactions. The steel rebar immersed in SP-2 solution exhibited pit formation during anodic scanning at 60 mV (*E_pit_*) on 0.97 mA (*i_pit_*). This result suggests that excess Cl^−^ ions broke the passive film, i.e., Zwitterion–Fe complex, and reached the steel rebar surface. The steel rebar immersed in SP-0 solution showed breakdown potential during anodic scanning, revealing the formation of metastable passive film.

Electrochemical parameters were extracted after the fitting of potentiodynamic polarization curves in Tafel slopes, and the results are illustrated in Table 4. This table shows that the corrosion potential (*E_corr_*) of the samples shifted towards the active direction once NaCl is added in solution. The *E_corr_* values of steel rebars immersed in SP-0, SP-1. and SP-2 solutions were found to be −363, −478, and −566 mV vs. SCE, respectively. This result suggests that the steel rebar immersed in SP-2 solution shows the corrosion of steel rebar [16,20,35,36,37]. The corrosion current densities (*i_corr_*) of steel rebar immersed in SP-0, SP-1, and SP-2 solutions are found to be 0.10, 0.45, and 1.96 µA·cm^−2^, respectively. Liu et al. [19] have suggested that if the *i_corr_* values are <0.1 µA·cm^−2^, then it is considered to be at passive levels and it is considered to be at low corrosion levels from 0.1 µA·cm^−2^ to <0.5 µA·cm^−2^. However, once the *i_corr_* value is within 0.5 to <1 µA·cm^−2^, then it is considered as severe corrosion. From the present studies, it can be concluded that the passive layer actually deteriorated with exposure to NaCl for SP-1, but it remains a stable layering in contrast to SP-2 where Cl^−^ ions ooze onto the passive film with time; therefore, the steel rebars immersed in SP-0, SP-1, and SP-2 solutions are observed to be at passive, low, and severe corrosion levels, respectively. Moreover, this finding revealed that 0.115 M LA containing steel rebar forms a passive film that can sustain up to 0.51 M NaCl during the breakdown of the passive film, but severe corrosion can occur once NaCl is increased up to 0.85 M at longer durations.

The corrosion rate (C.R.) of the samples immersed in different solutions is calculated by *i_corr_* values using the following equation [53], and the results are shown in Table 4:

(5) $$Corrosionrate\;(\mu m\cdot {\mathrm{year}}^{-1})=\frac{3.27\times i_{corr}\times E.W.}{d}$$

where *i_corr_* is the corrosion current density calculated by dividing the total exposed surface area of the steel rebar from the corrosion current; *E.W.* is the equivalent weight (g·mol^−1^); and *d* is the density (g·cm^−3^) of the Fe. The corrosion rates of steel rebar immersed in SP-0, SP-1, and SP-2 solution are found to be 1.16, 5.23, and 22.77 μm·year^−1^. The steel rebar immersed in SP-1 and SP-2 solutions exhibited almost 4.5 and 20 times higher corrosion rate compared to SP-0, respectively.

### 3.2. Characterization

#### Surface Morphology of Passive Film by SEM

**Figure 8:**
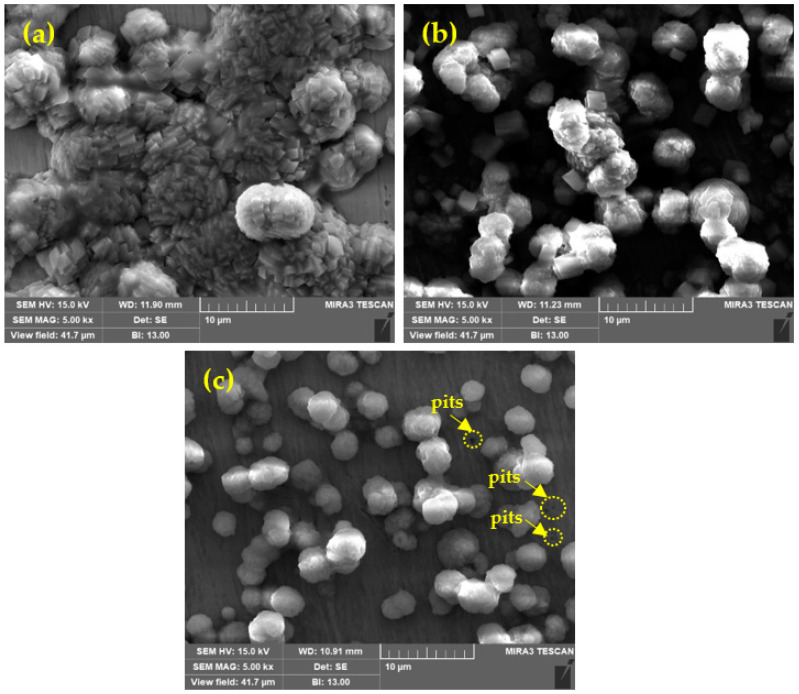
SEM after 168 h of immersion in (**a**) SP-0, (**b**) SP-1, and (**c**) SP-2 solutions.

The SEM images of passive films formed onto the steel rebar surface after 168 h of immersion in different solutions are shown in Figure 8. Figure 8a shows that the steel rebar immersed in SP-0 solution exhibited agglomeration in the passive film. Initially L-arginine (LA) was dissolved in the SP solution, but it started to react and form small Zwitterion–Fe complex once the steel rebar is immersed. As the exposure periods increased, most of the LA interacted with Fe and formed many Zwitterion–Fe complex clusters and adsorbed onto the steel rebar. Moreover, once 0.51 M NaCl was added in the SP + 0.115 M LA solution, i.e., SP-1, interaction between Cl^−^ ions with each Zwitterion–Fe complex was observed, resulting in the formation of the Cl-(Zwitterion)-Fe complex. Thus, the thickness of the passive film is greater than SP-0 (Table 3). This complex is adsorbed onto the rebar (Figure 8b). However, due to the smaller size of Cl^−^ ions remaining in the solution compared to the intermolecular pores of the adsorbed layer that permit slow propagation, the result is the initiation of corrosion, which is observed in Figure 7. Once the Cl^−^ ions interacted with the Zwitterion–Fe complex, the particles size of the cluster decreased and was regularly adsorbed; thus, dense morphology was observed (Figure 8b). Moreover, as NaCl increased up to 0.85 M in SP + 0.115 M LA, i.e., SP-2, and was exposed up to 168 h, the excess amount of Cl^−^ ions penetrated through the Cl-(Zwitterion)-Fe complex and initiated corrosion reactions; thus, pitting is observed in SEM (Figure 8c), and this result is well correlated with potentiodynamic polarization (Figure 7). As the NaCl amount increased, the particle size of the complex was reduced; thus, irregularity and vacancy between two particles are observed in Figure 8c.

EDS analyses of the passive film are shown in Table 5. The amount of Na and Cl increased once the NaCl amount increased in the solution. The Cl is found to be highest in the SP-2 solution, which revealed that Cl^−^ ions deposited onto the passive film resulted in highest corrosion rates. The results of Na, K, and Ca were a consequence of the composition of the pore solution where NaOH, KOH, and CaO were added. Ca was found in greater amounts compared to Na, and K is attributed to less dissolution of CaO and atmospheric carbonation. The presence of around 17–22% O revealed the formation of the passive film through the adsorption of LA molecules. The presence of around 1.5–2% N in all samples revealed that passive films contain LA, which formed a Zwitterion–Fe complex.

### 3.3. Plausible Mechanism for Initiation of the Corrosion Reaction

**Figure 9:**
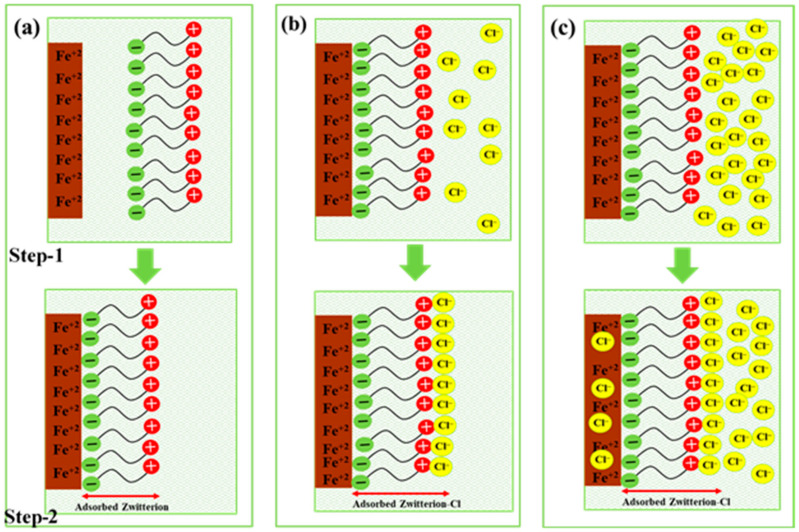
Schematic of (**a**) SP-0 for formation of Zwitterion–Fe complex as passive film, (**b**) interaction of Cl^−^ ions with passive film in SP-1, and (**c**) SP-2 solutions onto the steel rebar surface.

L-arginine (LA) is an amino acid that forms Zwitterion, i.e., protonated amino acid [27]. Amino acid can be used as inhibitor, and it is most effective when added with electronegative elements, i.e., halide ions [34]. A Zwitterion–Cl complex would form once the Cl^−^ ions are present in the solution. In LA containing SP solutions, the negative end of Zwitterion reacts with positively charged Fe metal and forms a Zwitterion–Fe complex, as shown in Figure 9a, up to 144 h of immersion. At higher immersion periods, the passive film, i.e., Zwitterion–Fe complex, strengthened the properties; thus, the highest impedance is observed. However, once 0.51 M NaCl was added into the solution, i.e., SP-1, the Cl^−^ ions tried to ingress through the passive film, but the positive end of Zwitterion interacted and formed Zwitterion–Cl complexes, as shown in step 1 of Figure 9b, which are adsorbed onto the steel surface (step 2 of Figure 9b) rather than being subject to corrosion. In this case, the Cl^−^ ions were limited and interacted with Zwitterion ions and formed Fe-(Zwitterion)-Cl/Cl-(Zwitterion)-Fe complexes; thus, protection is observed. Moreover, Cl^−^ ions observed in lesser amounts in SP-1 solution entered through the adsorbed Fe-(Zwitterion)-Cl complex, but once the amount of NaCl increased up to 0.85 M, i.e., SP-2, some amount of Cl^−^ ions interacted with Zwitterion and formed Zwitterion–Cl complexes. However, the excess amount of Cl^−^ ions penetrated through the adsorbed film and reached the steel rebar surface, which resulted in the initiation of corrosion reaction, as shown in Figure 9c. From this finding, it can be inferred that 0.115 M LA can sustain up to 0.51 M NaCl. The chloride threshold of LA in the present study is 0.51 M NaCl, which can protect steel rebars up to 168 h of exposure.

## 4. Conclusions

L-arginine is an eco-friendly corrosion inhibitor, which is adsorbed onto rebar surfaces and mitigates corrosion. In this study, a fixed amount 0.115 M LA has been used in an SP solution (SP-0) to initiate the growth of a passive film for 144 h. LA has been adsorbed onto the rebar by forming a Zwitterion–Fe complex. Once the proper passive film has been formed after 144 h, different amounts of NaCl, i.e., 0.51 M (SP-1) and 0.85 M NaCl (SP-2), are added in order to study the effect of Cl^−^ ions during the breakdown of the passive film and during the initiation of corrosion reactions. It can be observed from the present study that the steel rebar immersed in SP-0 solution exhibited the highest *R_p_* due to the formation of a protective passive film than compared to NaCl added to solutions with immersion periods. Once NaCl has been added in the solution, Cl^−^ ions initially interacted with the Zwitterion–Fe complex and formed a Cl-(Zwitterion)-Fe/Fe-(Zwitterion)-Cl complex, which protected the steel rebar; thus, the *R_ct_* value of steel rebar immersed in SP-1 and SP-2 solutions increased with exposure periods. However, the steel rebar immersed in SP-1 solution exhibited the highest *R_ct_* values compared to SP-2, and this is attributed to the formation of the Cl-(Zwitterion)-Fe complex as a protective passive film. Alternatively, SP-2 has the highest amount of NaCl where some amount of NaCl reacts with Zwitterion-Fe and formed a Cl-(Zwitterion)-Fe complex, but the excess amount of NaCl entered through the adsorbed layer/passive films and initiated corrosion reactions. However, the initiation of corrosion reaction requires some time in the SP-2 solution; thus, after 48 h of exposure, the OCP value shifted towards the active direction and the impedance values decreased dramatically. The potentiodynamic polarization results reveal severe corrosion as well as pits formation in SP-2 solution, while SP-0 exhibited immune/passive conditions and SP-1 shows low levels of corrosion after 168 h of NaCl addition. The chloride threshold of LA in the present study was found to be 0.51 M NaCl, which can protect steel rebars up to 168 h of exposure. The morphology of passive films by SEM reveals the formation of agglomerated and dense complex passive films in the SP-0 solution, while SP-2 shows pits and irregular morphology.

## Figures and Tables

**Table 1:** Chemical composition of steel rebar.

Elements (wt.%)										
Fe	Mn	Si	C	Cr	Ni	Cu	P	Mo	S	Sn
98.501	0.90	0.25	0.235	0.037	0.028	0.018	0.014	0.009	0.006	0.002

**Table 2:** Details of solution used in present study.

Sl. No.	Solution	Sample ID
1.	SP + 0.115 M LA	SP-0
2.	SP + 0.115 M LA + 0.51 M NaCl	SP-1
3.	SP + 0.115 M LA + 0.85 M NaCl	SP-2

**Table 3:** Electrochemical parameters of steel rebar immersed in different solutions.

Time (h)	Sample ID	Electrochemical Parameters							
		*R_s_* (Ω·cm^2^)	*CPE*_1_		*R_ct_* (kΩ·cm^2^)	*CPE_ct_*		Warburg (1 × 10^−3^) (Ω·cm^2^·s^0.5^)	*t_f_* (nm)
			*Q*_1_ (1 × 10^−5^) (Ω^−1^·cm^−2^·s*^n^*)	*n*_1_		*Q_ct_* (1 × 10^−5^) (Ω^−1^·cm^−2^·s*^n^*)	*n_ct_*		
1	SP-0	28 (±0.13)	10.3 (±0.06)	0.90 (±0.02)				4.66 (±0.27)	38.35 (±2.72)
	SP-1	14 (±0.08)	14.2 (±0.13)	0.81 (±0.02)	4.10 (±0.11)	12.5 (±1.25)	0.72 (±0.02)		45.02 (±2.97)
	SP-2	10 (±0.18)	17.2 (±0.76)	0.80 (±0.01)	1.20 (±0.04)	19.4 (±0.99)	0.62 (±0.01)		62.75 (±4.08)
48	SP-0	22 (±0.27)	13.0 (±0.15)	0.88 (±0.01)				7.13 (±0.63)	35.10 (±2.07)
	SP-1	12 (±0.10)	14.6 (±0.10)	0.81 (±0.01)	4.81 (±0.08)	11.1 (±0.08)	0.76 (±0.04)		60.90 (±3.84)
	SP-2	7 (±0.04)	19.8 (±1.56)	0.76 (±0.05)	1.24 (±0.11)	18.8 (±1.50)	0.63 (±0.04)		62.75 (±4.20)
168	SP-0	22 (±0.30)	11.9 (±0.78)	0.89 (±0.01)				3.79 (±0.45)	36.33 (±2.62)
	SP-1	13 (±0.01)	15.4 (±0.02)	0.80 (±0.05)	5.10 (±0.41)	10.6 (±0.02)	0.76 (±0.06)		64.71 (±3.56)
	SP-2	7 (±0.05)	20.2 (±0.03)	0.74 (±0.02)	2.07 (±0.01)	17.5 (±0.12)	0.65 (±0.01)		62.75 (±4.20)

**Table 4:** Electrochemical parameters of steel rebar.

Sample ID	Electrochemical Parameters			
	*E_corr_* (mV) vs. SCE	*i_corr_* (µA·cm^−2^)	Corrosion Level [19]	*C. R.* (µm·year^−1^)
SP-0	−363 (±1.25)	0.10 (±0.008)	Passive condition	1.16 (±0.009)
SP-1	−478 (±1.89)	0.45 (±0.05)	Low	5.23 (±0.52)
SP-2	−566 (±1.83)	1.96 (±0.15)	Severe	22.77 (±1.59)

**Table 5:** EDS analysis of passive film.

Sample ID	Elements (Wt.%)							
	O	Na	Cl	K	Ca	N	C	Fe
SP-0	21.87 (±3.82)	2.12 (±0.26)	-	0.42 (±0.12)	18.28 (±1.38)	2.04 (±0.22)	11.19 (±1.31)	Balance
SP-1	19.12 (±2.33)	3.64 (±0.28)	2.59 (±0.34)	0.56 (±0.07)	17.39 (±1.36)	1.94 (±0.19)	11.15 (±1.29)	Balance
SP-2	17.36 (±2.26)	3.71 (±0.35)	4.04 (±0.46)	0.32 (±0.09)	14.64 (±1.88)	1.57 (±0.22)	11.49 (±0.80)	Balance

## Data Availability

The raw/processed data required to reproduce these findings cannot be shared at this time as the data also forms part of an ongoing study.
